# Automated regional behavioral analysis for human brain images

**DOI:** 10.3389/fninf.2012.00023

**Published:** 2012-08-28

**Authors:** Jack L. Lancaster, Angela R. Laird, Simon B. Eickhoff, Michael J. Martinez, P. Mickle Fox, Peter T. Fox

**Affiliations:** ^1^Research Imaging Institute, The University of Texas Health Science Center at San AntonioSan Antonio, TX, USA; ^2^Institute of Neuroscience and Medicine (INM-1), Research CenterJülich, Germany; ^3^Institute for Clinical Neuroscience and Medical Psychology, Heinrich-Heine UniversityDüsseldorf, Germany

**Keywords:** BrainMap, Mango, behavior analysis, region of interest, brain atlas, TMS-PET, fMRI, ICA

## Abstract

Behavioral categories of functional imaging experiments along with standardized brain coordinates of associated activations were used to develop a method to automate regional behavioral analysis of human brain images. Behavioral and coordinate data were taken from the BrainMap database (http://www.brainmap.org/), which documents over 20 years of published functional brain imaging studies. A brain region of interest (ROI) for behavioral analysis can be defined in functional images, anatomical images or brain atlases, if images are spatially normalized to MNI or Talairach standards. Results of behavioral analysis are presented for each of BrainMap's 51 behavioral sub-domains spanning five behavioral domains (Action, Cognition, Emotion, Interoception, and Perception). For each behavioral sub-domain the fraction of coordinates falling within the ROI was computed and compared with the fraction expected if coordinates for the behavior were not clustered, i.e., uniformly distributed. When the difference between these fractions is large behavioral association is indicated. A *z*-score ≥ 3.0 was used to designate statistically significant behavioral association. The left-right symmetry of ~100K activation foci was evaluated by hemisphere, lobe, and by behavioral sub-domain. Results highlighted the classic left-side dominance for language while asymmetry for most sub-domains (~75%) was not statistically significant. Use scenarios were presented for anatomical ROIs from the Harvard-Oxford cortical (HOC) brain atlas, functional ROIs from statistical parametric maps in a TMS-PET study, a task-based fMRI study, and ROIs from the ten “major representative” functional networks in a previously published resting state fMRI study. Statistically significant behavioral findings for these use scenarios were consistent with published behaviors for associated anatomical and functional regions.

## Introduction

**Figure 1:**
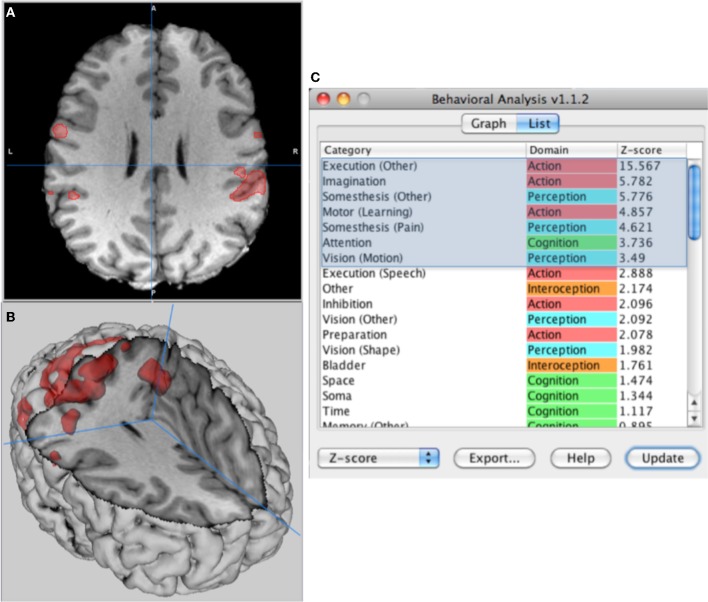
**Behavior analysis of left-hand finger tapping. (A)** High-resolution brain MRI with ROI from a functional MRI (fMRI) study, **(B)** surface rendering to illustrate the 3-D nature of the ROI, and **(C)** sub-domains listed by descending *z*-scores with statistically significant behaviors highlighted. Data can be viewed as a bar graph or exported as an Excel compatible file.

Relating findings from functional imaging studies to prior research is an important step in expanding our understanding of brain and behavior. Relevant publications are often found using keyword searches in databases such as Pub Med followed by *ad hoc* filtering, but interpretation can vary between researchers. Neuroimaging databases providing access to metadata from functional human brain research can help make more concise interpretations of behavior (Neurosynth—http://neurosynth.org/, Brede—http://neuro.imm.dtu.dk/services/jerne/brede/, PubBrain—http://www.pubbrain.org/, and BrainMap—http://www.brainmap.org/). However, finding relevant information in such databases can be difficult, the information is generally not presented in a manner that facilitates concise interpretation, and issues can arise regarding reverse inference (Poldrack, 2006, 2011). To address these problems we developed software to automate regional behavioral analysis of the human brain using data from the BrainMap database (http://www.brainmap.org/). The approach uses 3-D images formulated as spatial probability distributions of activation foci classified according to BrainMap's behavioral sub-domains. With over 20 years of development BrainMap has evolved into an extensive resource cataloging functional metadata from more than 2100 peer-reviewed papers, and over 10,000 experiments characterized using 83 paradigm classes. BrainMap categorizes functional imaging experiments using five major behavioral domains (action, cognition, emotion, interoception, and perception) with 51 sub-domains (Fox et al., 2005; Table 1). Each experiment is assigned one or more behavioral classifications along with a set of *x*-*y*-*z* coordinates for reported activations, and these data provide the basic structure for forming behavioral probability distributions as 3-D images. Region of interest (ROI) analysis is applied to these spatial probability images to assess behaviors. Findings can be charted as a “behavior profile” or viewed as *z*-score significance ranked behavior listing (Figure 1) to facilitate interpretation. The variety of experiments, imaging systems, processing methods, and paradigm classes in the BrainMap database provide breadth and depth for behavioral analyses.

**Table 1:** **BrainMap behavior categorization by domain and sub-domain**.

**Action**	**Cognition**	**Emotion**	**Interoception**	**Perception**
1. Execution:Other (8518)	10. Attention (10,995)	27. Anger (507)	35. Air-hunger (236)	43. Audition (2850)
2. Execution:Speech (3399)	11. Language:Orthography (2011)	28. Anxiety (577)	36. Bladder (315)	44. Gustation (1173)
3. Imagination (1244)	12. Language:Other (1204)	29. Disgust (879)	37. Hunger (386)	45. Olfaction (400)
4. Inhibition (2519)	13. Language:Phonology (1621)	30. Fear (1311)	38. Other (200)	46. Somethesis (2542)
5. Motor:Learning (832)	14. Language:Semantics (7593)	31. Happiness:Humor (120)	39. Sexuality (877)	47. Somethesis:Pain (3472)
6. Observation (972)	15. Language:Speech (7244)	32. Happiness (1060)	40. Sleep (260)	48. Vision:Color (201)
7. Other (11)	16. Language:Syntax (655)	33. Other (12,821)	41. Thermoregulation (29)	49. Vision:Motion (2514)
8. Preparation (346)	17. Memory:Explicit (7002)	34. Sadness (1167)	42. Thirst (209)	50. Vision:Other (2106)
9. Rest (1611)	18. Memory:Other (50)			51. Vision:Shape (2995)
	19. Memory:Working (7819)			
	20. Music (822)			
	21. Other (8847)			
	22. Reasoning (1387)			
	23. Social (1562)			
	24. Soma (581)			
	25. Space (1935)			
	26. Time (495)			
Total (19,452)	Total (61,783)	Total (18,442)	Total (2512)	Total (18,253)

The number of activation foci in the brain for each sub-domain () along with totals by domain are provided.

Behavioral analysis software was developed and tested as a plugin application for the Multi-image Analysis GUI (Mango) image processing system (http://ric.uthscsa.edu/mango/). Several features of Mango were important in this development: (1) ease of use, (2) multi-platform Java application, (3) extensive ROI tools, (4) ability to add and update software as a plugin module and (5) full access to a suite of image viewing and processing features. Mango is available from the Neuroimaging Tools and Resources (NITRC) website (http://www.nitrc.org/) and the Research Imaging Institute (RII) website (http://ric.uthscsa.edu/mango/). Regular updates for Mango have been provided with additional features (12 releases through 2012), and over 10,000 copies of Mango have been downloaded.

A primary goal of the automated “behavioral analysis” project was to provide software that would rapidly determine regionally specific behaviors for researchers' brain studies. Summing activation foci within an ROI for each behavioral sub-domain was the initial step; however, additional processing was necessary to properly gauge the relationship between these data and BrainMap's behavior domains. The next processing step was to convert ROI sums to activation probabilities. Further processing was done to correct probabilities for region size effects. Finally, statistical validity was provided as *z*-scores testing the null hypothesis that the distribution for activation foci observed within an ROI was not different from that predicted for a spatially uniform random distribution within the brain. This paper describes development methods, characteristics, and provides use scenarios for behavioral analysis with functional and anatomical images.

## Methods

Each experiment in the BrainMap database is behaviorally classified using one or more sub-domains. Behaviors that classify well by major domain but do not match an existing sub-domain are classified as Domain:Other, such as “Action:Other” in Table 1. Similarly, behaviors that classify well by sub-domain but do not match other sub-domains are classified as Domain:Sub-domain:Other, such as “Perception:Vision:Other”. This provides completeness for classification of experiments by domain and sub-domain. The “Other” groupings may subsequently be subdivided and designated more explicitly as the BrainMap database continues to grow.

**Figure 2:**
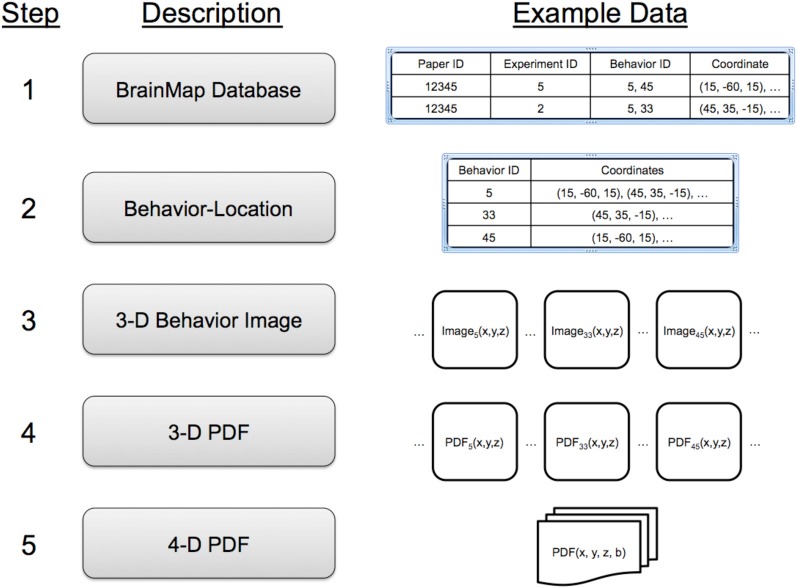
**A five-step process is used to extract coordinate and behavior data from the BrainMap database and formulate a behavioral probability density function (PDF) as a 4-D image indexed using *x*-*y*-*z* coordinates and behavior (b)**.

**Figure 3:**
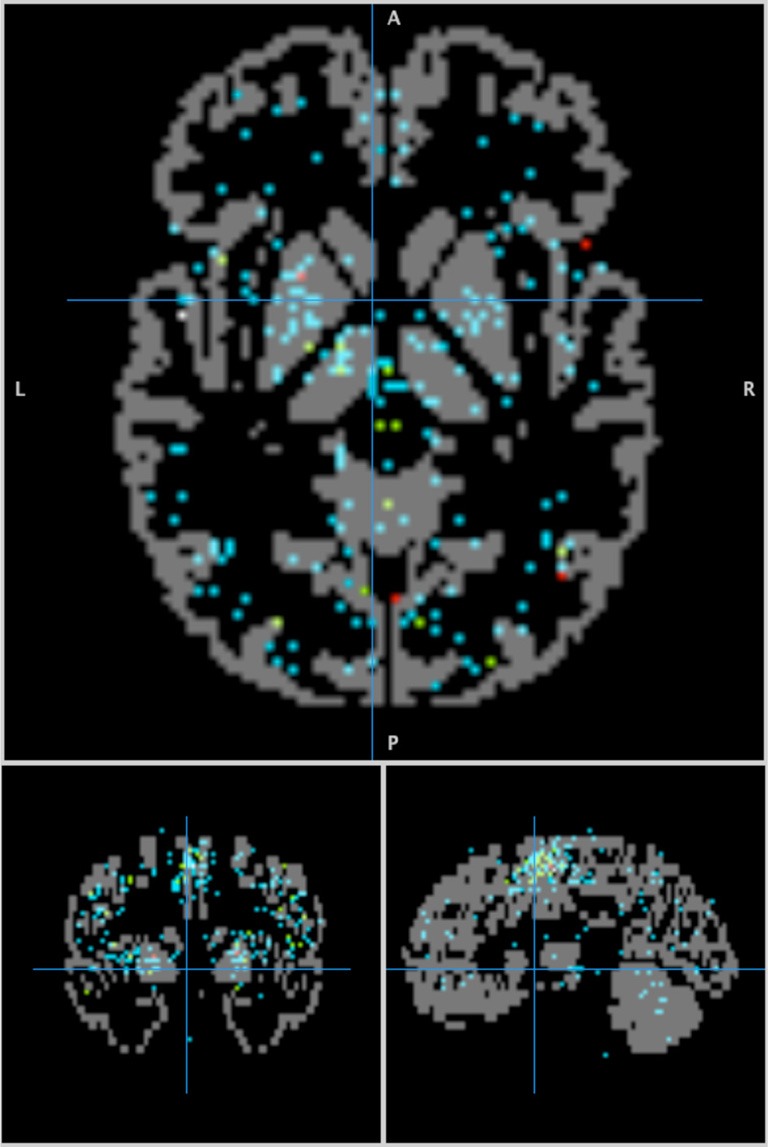
**The ‘Action:Execution’ behavior sub-domain image.** Activation foci are overlaid onto gray matter from the Talairach Daemon (Lancaster et al., 2000) to provide an anatomical background. Crosshair at (−4, 0, 0).

A five-step process was used to make a data structure to rapidly index locations and behaviors (Figure 2). In Step 1 (BrainMap Database), each experiment in the BrainMap database is isolated, and Talairach coordinates for the experiment's activation locations (activation foci) recorded. Experiments often have multiple Behavior IDs to cover the multiple behaviors involved. In Step 2 (Behavior-Location), data are reorganized as a table of behavior sub-domains (Behavior IDs) with a list of coordinates for each. Note that the same coordinate can be associated with more than one Behavior ID. In Step 3 (3-D Behavior Image), a 3-D image of activation foci is formulated for each behavioral sub-domain. Images were formatted with 2-mm isotropic spacing, similar to the spatial precision in functional brain images, with locations indexed by Talairach coordinates. For each location in a behavior sub-domain's coordinate list we added “one” to its image, such that the resulting image tabulated activation foci by location. An example of an activation foci image formed in this manner is illustrated for the “Action:Execution” sub-domain in Figure 3. In Step 4 (3-D PDF), activation foci images are converted to 3-D probability density function images PDF(*x*, *y*, *z*) by dividing each by the total number of activation foci (*N*_*b*_) in the brain for its sub-domain (Table 1). Finally, in Step 5 (4-D PDF), the set of 51 3-D PDF images were concatenated into a single 4-D probability density image PDF(*x*, *y*, *z*, *b*) where location and behavior can be readily indexed. This 4-D image is stored using the NIH's NIfTI (Neuroimaging Informatics Technology Initiative) file format (http://nifti.nimh.nih.gov) with gzip compression (http://www.gzip.org/) for efficient distribution.

The regional probability for each sub-domain's behavior (b) is determined by summing PDF(*x*, *y*, *z*, *b*) over the *x*-*y*-*z* extent of a brain ROI. These probabilities range from near zero for a small ROI to unity for a whole-brain ROI. The probability for an ROI varies by sub-domain according to location, size and shape and increases as the ROIs spatial configuration approaches that of the activation foci in a behavior sub-domain. PDF-based behavior images rather than Activation Likelihood Expectation (ALE) based behavior images (Laird et al., 2009) were used to provide a direct means to calculate probabilities.

Automated behavior analysis requires that brain images be spatially normalized. Internal calculations use Talairach coordinates (Lancaster and Fox, 2009). A built-in MNI-to-TAL affine transform (Jenkinson and Smith, 2001; Lancaster et al., 2007) is provided to adjust images fitted to the Montreal Neurological Institute (MNI) brain space (Evans et al., 2012) to the Talairach brain space. Images from popular functional image analysis software applications such as FMRIB Software Library (FSL http://www.fmrib.ox.ac.uk/fsl/), Statistical Parametric Mapping (SPM http://www.fil.ion.ucl.ac.uk/spm/) and Analysis of Functional Neuroimages (AFNI http://afni.nimh.nih.gov/afni/) are supported using the NIfTI file format. ROI tools in Mango can be used to threshold statistical parametric images (e.g., *z*-scores) to provide 3-D ROIs for behavior analysis. Probabilities for an ROI are determined for the 51 behavioral sub-domains, and when organized by domain: sub-domain provides a “behavior profile.” Probabilities are the probability that reported behavior-specific activation foci fell within the ROI. The use of probabilities, rather than activation foci sums, controls for the differences in numbers of foci per sub-domain (Table 1).

The measured or “observed” probability *p*_*o*_ increases as ROI size increases reaching unity for a whole brain ROI. We therefore sought a size-adaptable expected probability (*p*_*e*_) for significance testing. Like observed probability the “expected” probability needed to range from zero to unity as ROI volume changed from zero to full brain volume, but not vary by location or shape. To meet these needs *p*_*e*_ was calculated as the ROI-to-brain volume ratio, with brain volume determined from the Talairach Daemon (Lancaster et al., 2000), which is sized according to the 1988 Talairach Atlas brain (Talairach and Tournoux, 1988). The expected probability *p*_*e*_ is therefore an estimate of observed probability *p*_*o*_ should activation foci for a sub-domain be randomly distributed throughout the brain, i.e., not regionalized.

To test for significance of behaviors we used the null hypothesis that the observed probability of activation foci was not different from expected, i.e., that *p*_*o*_ = *p*_*e*_. This test was done for each of the 51 behavior sub-domains. The difference (*p*_*o*_ − *p*_*e*_) is called the effect size, and when it is positive more activation foci are seen within the ROI than expected for random spatial distribution. Likewise when the effect size is negative fewer activation foci are seen than expected for random spatial distribution. The focus for behavior analysis is therefore on positive effect sizes. To determine variance for effect size we modeled the two possible outcomes of activations (inside or outside of the ROI) using the binomial distribution. In this study *p*_*o*_ and *p*_*e*_ served as binomial “success” probabilities (probability of activations falling within the ROI), and the number of trials was the whole-brain activation tally (*N*_*b*_) for a sub-domain. For the binomial distribution the variance of “*p*” is calculated as *p*(1-*p*)/*N*. An effect-size *z*-score for each behavioral sub-domain was calculated as follows:

(1) $$z=\frac{p_{o}-p_{e}}{{\left(\frac{p_{o}(1-p_{o})+p_{e}(1-p_{e})}{N_{b}}\right)}^{1/2}}$$

Only behavioral sub-domains with positive *z*-scores ≥ 3.0 are considered significant (Bonferroni corrected to overall *p*-value of 0.05 for the 51 behavior sub-domains. Results for each sub-domain are provided as total foci, observed probability (*p*_*o*_), relative probability (*p*_*o*_ − *p*_*e*_)/*p*_*e*_, and *z*-scores. These are viewable as charts and a table of ranked values to facilitate interpretation.

### Processing times

The processing speed for behavior analysis software with the 2-mm spacing 4-D PDF image was tested on two systems using the ROI from Figure 1, (1) a windows based desktop PC (Sony Vaio PCV- RZ32G) with an Intel Pentium 4 (2.6 GHz) processor running Windows XP (SP3) and (2) a MacBook Pro with Intel core duo processor (2.33 GHz) running OS X 10.6.7. The PC had 1.5 GB of RAM and the Mac had 2 GB of RAM, and a faster internal bus. Both systems performed analysis and updated results within 1 s for the ROI illustrated in Figures 1A,B. Processing speed varies with ROI size, but even with hemisphere size ROIs processing time was ~1 s on the MacBook Pro. Application startup time was 1.3–2.5 s mostly due to initialization of the 4-D PDF image. Processing times were increased by 4–8X for a 1-mm 4-D PDF image. These tests show that behavior analysis results are available almost immediately and facilitate rapid interpretation and exploration.

### Spatial precision of the PDF image

Most functional images are acquired with relatively low spatial precision (sample spacing >2 mm), so ROIs derived from these images are also considered low resolution. The 4-D PDF image was made using 2-mm spacing for analysis of such low-resolution images. However, behavior analysis can also be done using ROIs from high spatial resolution anatomical images (~1 mm spacing), so we made a 1-mm spacing 4-D PDF image to evaluate this use. We tested both low- and high-resolution images using both 1- and 2-mm spatial precision PDFs. The behavior analysis software down converts 1-mm precision ROIs to 2-mm precision for use with the 2-mm spacing PDF image and up converts 2-mm ROIs to 1-mm precision for use with the 1-mm spacing PDF image.

High-resolution ROI testing used spherical ROIs from a brain image with 1-mm spacing. High-resolution ROIs were defined as spheres of 12-mm radius positioned at two brain sites of interest, the supplementary motor area (SMA) at Talairach co-ordinate (−1, 4, 48) and the anterior cingulate (−1, 43, −1). Low-resolution ROI testing (~2 mm spacing) was done using an ROI made by thresholding an individual fMRI study at *z*-score = 2.5. The ROI was from the fMRI study illustrated in Figure 1.

### Symmetry of activation foci

To examine the balance of foci reported in left and right brain we evaluated the left-right (L-R) symmetry of activation foci in the brain. Tallies of activation foci for left and right brain were made from the 100,000+ locations reported in BrainMap, and a *z*-score determined for the fraction of activation foci left of midline. Analysis was done for major anatomical subdivision of the brain by hemispheres and by lobes. Further symmetry analysis was done by behavior evaluating each of the 51 behavior sub-domain images. Finally, symmetry analysis was performed for the language areas, Broca's (BA44 and 45) and Wernicke's (posterior BA22), which favor the left hemisphere. ROIs for these language areas were based on their Brodmann Areas defined in the 1988 Talairach atlas. The posterior portion of BA22 was isolated using *y*-coordinates posterior of *y* = −27. The two language-area ROIs were enlarged to help account for spatial and anatomical variability by dilating twice using a 3×3×3 kernel.

### Use scenarios

Since “behavior analysis” is co-ordinate based it can be used for analysis of images where the brain is registered to the MNI or Talairach brain spaces. This allowed us to provide use scenarios over a wide range of interests including a brain atlas, a TMS/PET study, a task-based fMRI study and a published resting state network study.

#### Harvard-oxford cortical (HOC) atlas

The brain atlases distributed with the FSL software (http://www.fmrib.ox.ac.uk/fsl/data/atlas-descriptions.html) have well defined anatomical regions delineated by numeric values so that they can be readily defined as ROIs. We selected eight gyral regions from the HOC atlas for behavioral analysis (http://www.cma.mgh.harvard.edu/fsl_atlas.html). The eight regions spanned from the middle frontal gyrus (MFG) to the occipital pole (OP). The 1-mm 25% thresholded maximum probability atlas was used for this study. The HOC atlas is in MNI space so we applied the MNI-to-TAL transform before processing. ROIs were defined using the numeric values designated for each of the eight brain regions.

#### TMS/PET study

Behavior analysis was used by (Narayana et al., 2012) to compare behaviors associated with SMA connected regions using connectivity determined by stimulation based TMS/PET (Fox et al., 1997, 2006; Paus et al., 1997; Laird et al., 2008) and by meta-analytic connectivity modeling (MACM) (Fox et al., 1998; Robinson et al., 2010; Cauda et al., 2011; Eickhoff et al., 2011; Torta and Cauda, 2011). Statistical parametric maps for MACM and TMS/PET studies were formulated based on brain areas co-varying with right SMA. ROIs delineating significant brain regions (*z* ≥ 3) were used as input for behavior analysis.

#### Task-based functional MRI (fMRI) study

A statistical parametric image indicating active brain areas from a fMRI study was used to demonstrate behavioral analysis for a single subject. The task was a finger tapping sequence of the non-dominant (left) hand used in a motor learning project (Figure 1). The statistical parametric image was formatted as a 2×2×2 mm *z*-score image aligned to the Talairach brain space. A single ROI was formulated using a *z*-score threshold of *z* = 2.5. Behavior analysis was done using this ROI.

#### Resting state networks

Functionally connected regions within the brain are identifiable using resting state fMRI and independent component analysis (ICA) (Arfanakis et al., 2000; Bartels and Zeki, 2005; Ma et al., 2007; Jafri et al., 2008). Smith et al. (2009) published their fMRI-ICA findings from a resting-state study of 36 subjects. They performed an ICA analysis using a model order of 20 and converted the ICA spatial maps to *z*-statistic images, then thresholded using *z* ≥ 3 to isolate components as regions. Ten of these components were considered as the “major representative” functional networks, based on remarkable correspondence observed between components derived from ICA of resting state fMRI data and those from BrainMap-derived ICA components of co-activating networks. They provided detailed descriptions of associated behaviors for the ten networks, as determined from extensive review of the BrainMap database. We downloaded these regions from the FMRIB website (http://fsl.fmrib.ox.ac.uk/analysis/brainmap+rsns/) and performed behavior analysis for each of the ten components for comparison with author's behavioral descriptions.

## Results

### Spatial precision of the PDF image

Behavior analysis for the high-resolution spherical ROI in SMA indicated six significant sub-domains for the 1-mm behavior image and seven significant sub-domains for the 2-mm behavior image. The slight mismatch occurred for a sub-domain where the *z*-score was near the significance threshold (*z* = 3.0). Behavior analysis for the high-resolution spherical ROI in anterior cingulate indicated five significant behavioral sub-domains using the 1-mm behavior image and four with the 2-mm behavior image; again the mismatch was where the *z*-score was near the significance threshold value.

Similar results were seen for the low resolution ROI derived from the fMRI study with 6 of 7 matching significant sub-domains. A paired *t*-test was performed comparing the *z*-score behavior profiles for 1-mm and 2-mm 4-D PDF images and for 1-mm and 2-mm precision ROIs, and all *p*-values were less than 0.03. Behavioral sub-domains with *z*-scores >4.0 were identical regardless of the precision in forming ROIs (1-mm or 2-mm) or precision used for the 4-D PDF image. The small differences in behavioral analysis results should have minimal effect for automated behavior analysis where the variability in ROI position, size, and shape are more important. Based on these results we opted to use the 2-mm 4-D PDF image with the behavior analysis software.

### Symmetry of activation foci

A small but highly statistically significant leftward fraction (54%) was seen for the cerebrum (Table 2). The distribution within the cerebellum was slightly rightward (51%) but not statistically significant. Significant asymmetry was seen in all cerebral lobes. In three lobes (Frontal, Temporal, and Parietal) there was a large leftward trend (54–55%). In the Occipital and sub-Lobar regions the leftward trend was smaller (52%) with lesser *z*-scores. Finally, the Limbic lobe had the smallest leftward trend (51%), which was only slightly above the threshold for statistical significance.

**Table 2:** **Left-Right distribution of activation foci by brain region**.

**Region**	**Volume (mm^3^)**	**Left fraction**	***z*-score**
Cerebrum	1,310,229	0.54	22.9^*^
Cerebellum	159,554	0.49	−1.6
Frontal lobe	474,393	0.55	18.7^*^
Temporal lobe	216,674	0.54	9.4^*^
Parietal lobe	180,664	0.54	10.2^*^
Occipital lobe	143,634	0.52	4.0^*^
Limbic lobe	120,585	0.51	2.3^*^
Sub-lobar	165,115	0.52	4.4^*^

* Significant z-scores (|z| ≥ 2.0).

Only 13 of the behavior sub-domains (~25%) indicated a statistically significant L-R difference (Table 3). One sub-domain “Action:Inhibition” had a rightward trend (55%). A large leftward trend was seen for language related sub-domains with left fractions of 60% or more. No statistically significant asymmetry was seen for the Emotion and Interoception domains. The leftward trend for “Action:Execution” was likely due to the fact that most hand related tasks are performed with the right hand.

**Table 3:** **Left-Right distribution of activation foci by behavior sub-domain**.

**Sub-domain**	**Domain**	**# Foci left**	**# Foci right**	**L + R**	**Left fraction**	**Left *z*-score**
Execution:Other	Action	3924	3270	7194	0.55	7.7^*^
Execution:Speech	Action	1625	1254	2879	0.56	7.0^*^
Imagination	Action	645	454	1099	0.59	5.9^*^
Inhibition	Action	1036	1254	2290	0.45	−4.6^**^
Language:Orthography	Cognition	1050	662	1712	0.61	9.6^*^
Language:Other	Cognition	660	415	1075	0.61	7.7^*^
Language:Phonology	Cognition	907	525	1432	0.63	10.5^*^
Language:Semantics	Cognition	4232	2349	6581	0.64	24.2^*^
Language:Speech	Cognition	3829	2523	6352	0.60	16.7^*^
Language:Syntax	Cognition	392	196	588	0.67	8.6^*^
Memory:Explicit	Cognition	3459	2736	6195	0.56	9.2^*^
Memory:Working	Cognition	3591	3311	6902	0.52	3.4^*^
Somesthesis:Other	Perception	1157	1009	2166	0.53	3.2^*^

* Significant leftward.

** Significant rightward (|z| ≥ 3.0).

Behavioral analysis for the language ROIs indicated significant language behaviors in the left hemisphere, “semantics” and “speech”. No significant behaviors were indicated for the right-side ROIs. These results are consistent with left dominance of language for Broca's and Wernicke's areas. The observed L-R symmetry of activation foci was consistent with the expected symmetries (Banich, 2004), with a slight overall leftward trend primarily due to the dominance of language areas on the left.

### Use scenarios

#### HOC atlas

Significant behaviors (*z*-score = 3.0) were seen for each of the eight anatomically defined brain regions (highlighted in Table 4). The summary of major findings organized by behavioral domain is as follows:

**Table 4:** **Behavior analysis of eight anatomical regions from the HOC atlas**.

**Sub-domain**	**Domain**	**SFG**	**MFG**	**preCG**	**postCG**	**SPL**	**LOCS**	**LOCI**	**OP**
Execution (Other)	Action	2.73	−0.41	22.37	14.80	11.34	4.11	−1.08	−3.30
Execution (Speech)	Action	−0.82	1.19	9.11	3.78	−0.15	−3.41	−0.65	2.69
Imagination	Action	1.86	2.49	8.11	2.83	5.11	1.77	−0.50	−2.59
Inhibition	Action	4.18	6.64	1.30	−1.52	3.02	5.45	1.44	−1.05
Motor (Learning)	Action	3.44	1.90	4.31	2.94	3.88	2.35	−0.42	−1.97
Observation	Action	1.00	2.97	2.43	0.17	4.78	2.69	6.40	1.67
Other	Action	0.69	−0.51	0.53	−0.57	−0.37	−0.68	−0.44	−0.49
Preparation	Action	1.63	1.64	1.96	1.05	0.84	0.38	1.26	−0.12
Rest	Action	−3.35	3.75	−0.60	−1.24	−1.99	3.02	−1.08	−1.70
Attention	Cognition	5.36	12.76	5.07	−1.75	7.94	10.87	4.06	−0.91
Language (Orthography)	Cognition	1.61	1.99	3.50	−0.52	2.99	4.38	6.14	5.24
Language (Other)	Cognition	0.73	3.19	2.81	−1.23	1.22	3.01	2.98	3.57
Language (Phonology)	Cognition	0.87	5.29	4.75	−2.41	0.50	1.40	−0.07	0.36
Language (Semantics)	Cognition	2.77	8.37	3.36	−4.17	1.65	1.78	7.21	3.39
Language (Speech)	Cognition	0.63	6.55	7.92	−1.01	1.01	−1.04	2.55	2.95
Language (Syntax)	Cognition	−1.08	1.69	1.87	−2.33	0.48	1.83	−1.54	−0.63
Memory (Explicit)	Cognition	4.26	9.14	0.71	−5.43	2.95	5.99	2.37	−1.20
Memory (Other)	Cognition	−0.12	0.48	0.56	−0.28	−0.78	−0.01	0.69	−0.05
Memory (Working)	Cognition	8.18	17.25	7.54	−3.55	7.53	11.80	2.21	−0.99
Music	Cognition	1.54	0.44	2.34	0.64	2.70	−1.38	−2.37	−1.70
Other	Cognition	4.31	6.51	−0.39	−4.23	3.72	3.76	0.73	−1.49
Reasoning	Cognition	3.08	7.74	−0.86	−2.12	3.32	7.10	0.07	0.88
Social	Cognition	4.01	1.57	0.61	−2.51	0.16	2.76	0.47	−1.28
Soma	Cognition	1.11	1.44	2.04	−0.67	2.81	1.55	2.61	0.48
Space	Cognition	3.67	3.93	3.60	−1.69	6.97	13.12	6.35	0.78
Time	Cognition	1.97	2.93	1.80	−1.08	1.22	0.77	0.14	1.22
Anger	Emotion	−0.19	2.35	1.18	−2.17	−1.47	−0.24	0.75	−1.81
Anxiety	Emotion	0.97	−0.30	0.77	−1.73	−0.91	−0.51	0.27	−0.99
Disgust	Emotion	−0.78	0.38	−2.32	−2.79	−2.45	0.19	3.12	1.07
Fear	Emotion	−0.65	0.78	−0.58	−4.22	−1.40	−2.31	4.42	−0.03
Happiness (Humor)	Emotion	0.46	−0.37	−0.94	−1.17	−0.29	0.05	1.94	−1.63
Happiness (Other)	Emotion	−0.13	0.58	1.07	−2.12	−2.50	−1.92	2.93	−0.44
Other	Emotion	3.84	5.74	−2.12	−6.10	0.39	−0.42	2.86	−2.41
Sadness	Emotion	−0.20	2.33	0.09	−3.02	−1.09	−2.51	1.59	1.04
Air-hunger	Interoception	−1.30	−0.16	−0.97	−1.62	−1.71	−3.15	−0.38	−1.67
Bladder	Interoception	−0.11	−1.01	2.88	−0.50	−1.30	−2.17	−2.33	−2.63
Hunger	Interoception	0.43	−1.75	−0.82	−0.96	−1.56	−1.83	2.96	−0.34
Other	Interoception	0.68	0.41	3.59	1.53	0.62	−1.19	−1.86	−2.10
Sexuality	Interoception	−0.95	−0.41	−0.62	−0.92	1.38	1.52	5.96	0.64
Sleep	Interoception	1.98	−0.02	−1.84	−1.79	1.47	1.94	0.50	−1.32
Thermoregulation	Interoception	0.25	−0.83	−1.04	−0.92	−0.60	−1.10	−0.71	1.31
Thirst	Interoception	−0.69	0.34	−0.73	1.46	−0.26	−0.95	−1.20	−0.99
Audition	Perception	−0.82	3.72	1.93	0.05	1.27	−1.04	−3.31	−3.69
Gustation	Perception	−0.22	0.85	1.21	0.31	−1.10	−1.03	1.18	1.51
Olfaction	Perception	−0.60	0.98	−2.18	−1.60	0.04	−0.79	−1.22	−0.69
Somesthesis (Other)	Perception	−0.74	0.60	8.19	8.67	4.80	−1.70	−2.22	−1.53
Somesthesis (Pain)	Perception	0.94	0.15	3.41	1.89	1.49	−5.76	−4.56	−7.11
Vision (Color)	Perception	0.39	2.74	−0.35	−1.85	2.19	1.84	2.24	4.45
Vision (Motion)	Perception	4.71	2.60	7.46	−2.30	8.31	11.86	6.31	3.32
Vision (Other)	Perception	1.20	4.24	3.98	−2.61	2.93	7.15	4.92	5.43
Vision (Shape)	Perception	1.57	2.89	3.30	−2.41	6.05	11.19	11.06	5.44

Significant behaviors (z≥3.0) highlighted

SFG, superior frontal gyrus; MFG, middle frontal gyrus; preCG, pre central gyrus; postCG, post central gyrus; SPL, superior parietal loblule; LOCS, lateral occipital cortex superior; LOCI, lateral occipital cortex inferior; OP, occipital pole

***Action.*** Significant behaviors were seen for all regions except the OP. Significant findings for Execution (Speech) were only seen in the precentral and postcentral gyri. The largest *z*-scores were for Execution (Other) in the precentral gyrus (preCG), postcentral gyrus (postCG), and superior parietal lobule (SPL). Action execution is often a necessary part of task-based functional imaging studies, and this was reflected by the largest overall *z*-score in the preCG. Two action sub-domains were not significant in any of the eight brain regions, “Other” and “Preparation.”

***Cognition.*** Significant behaviors were seen for all regions except the postCG. The two largest *z*-scores were in the MFG for “working memory” and in lateral occipital cortex-superior (LOCS) for “Space.” The major behavior sub-domains for the two frontal regions (SFG and MFG) were “working memory” and “Attention.” The only region with a significant *z*-score for the “Social” sub-domain was the superior frontal gyrus (SFG).

***Emotion.*** Only three brain regions (SFG, MFG, and lateral occipital cortex-Inferior (LOCI) had significant emotion *z*-scores. For the LOCI region the behaviors were “Disgust” and “Fear” while for the frontal regions behavior was non-specific (Other).

***Interoception.*** Similar to the Emotion domain, only two regions had significant *z*-scores, preCG and LOCI. For the LOCI region the behavior sub-domain was “Sexuality” while the preCG was non-specific (Other).

***Perception.*** Significant perception behaviors were found in all eight regions. Many regions indicated significant visual involvement, with largest *z*-scores for the three occipital regions. The only region with a significant *z*-score for “color vision” was the OP. None of the eight brain regions indicated “Gustation” or “Olfaction” as a significant behavior.

The associations between brain regions and behaviors followed general expectations, with language mostly in the MFG and preCG and vision mostly in the occipital regions (Banich, 2004).

#### TMS/PET study

MACM and TMS/PET statistical parametric images indicated similar SMA connectivity patterns, with the MACM connections being more extensive (Narayana et al., 2012). Behavior analysis of MACM regions revealed significant behaviors for all major and numerous minor sub-domains. Behavior analysis of the TMS/PET regions indicated significant behaviors in fewer sub-domains, with no behaviors reported for the Interoception domain. However, the largest *z*-scores for TMS/PET in Action, Cognition, Emotion, and Perception domains matched those for MACM. The author's concluded that MACM informed on the broad functional nature of SMA connections, while TMS/PET identified the more specific electrophysiological connectivity of SMA, and importantly behavioral analysis mirrored this finding with broad vs. restricted behavioral findings.

#### Task-based fMRI study

The ROI for the fMRI study encompassed brain areas that are assumed to be active in a finder-tapping study (large right M1 region, a small left M1 region, a SMA region, and smaller regions in left cerebellum; see Figures 1A,B). The behavioral listing (Figure 1C) indicated distinct significant behaviors associated with Action, Perception, and Cognition domains, with statistically significant behavior sub-domains highlighted. The first five of these are typical for a motor learning task. The sixth “Perception:Somesthesis (Pain)” might have been related to the experience of performing the task in an MRI scanner. The most significant behavior was “Action:Execution” (*Z* = 15.57). The high *z*-score for this behavior is an indication of the large fraction of activation foci from the “Action:Execution” behavior sub-domain (Figure 3) within the ROI, especially the components in M1 and SMA. These results indicate high specificity of behavior analysis for an individual fMRI study when the task is carefully controlled.

#### Resting state networks

Flagged significant behavior sub-domains (*z* ≥ 3.0) for the 10 resting state networks ICA1-10 (Table 5) matched well with their published descriptions (Smith et al., 2009). A full functional explication based on examination of BrainMap metadata has been provided for these 10 networks (Laird et al., 2011). The results observed from the fully automated behavioral analysis generally agree with these prior works. Specifically, we observed a strong correspondence between the default mode networks (ICA4) and the domains of social cognition, explicit memory, and rest, as well as a lack of domain prevalence for the cerebellar network (ICA5), indicating the functional heterogeneity of this brain region.

**Table 5:** **Behavioral analysis of ten major representative functional networks (Smith et al., 2009)**.

**Sub-domain**	**Domain**	**ICA1**	**ICA2**	**ICA3**	**ICA4**	**ICA5**	**ICA6**	**ICA7**	**ICA8**	**ICA9**	**ICA10**
Execution (Other)	Action	−3.56	−2.55	3.47	−11.36	3.93	37.19	2.00	−4.28	−7.13	−0.38
Execution (Speech)	Action	−0.88	1.29	1.97	−4.97	3.44	10.16	14.13	−1.59	−6.77	−0.35
Imagination	Action	−1.08	−2.83	1.92	−1.60	−0.08	11.41	0.37	0.34	−2.79	5.31
Inhibition	Action	−1.88	−2.99	1.43	−0.70	−6.29	3.63	3.20	10.88	5.16	3.63
Motor (Learning)	Action	−0.32	−1.38	3.20	−0.51	1.47	8.36	−0.09	0.80	−2.30	0.87
Observation	Action	0.68	1.11	7.67	−3.44	−2.89	1.93	−0.75	−0.23	0.56	3.37
Other	Action	−0.91	−0.77	0.02	0.15	−0.82	0.03	0.75	0.37	−0.08	0.58
Preparation	Action	−0.66	−0.67	0.43	0.89	−2.09	4.28	−0.32	2.67	−0.26	−0.17
Rest	Action	−2.45	−3.59	−2.50	7.08	−3.89	−2.91	0.47	4.73	0.57	3.82
Attention	Cognition	0.68	−1.99	8.92	−2.55	−8.63	8.84	4.02	12.17	2.39	10.84
Language (Orthography)	Cognition	−0.06	5.83	10.67	−2.82	−0.85	1.53	0.17	−1.18	−4.79	6.75
Language (Other)	Cognition	−0.69	2.24	4.69	−0.62	−1.94	1.35	3.64	−2.23	−3.17	5.31
Language (Phonology)	Cognition	−3.49	−0.23	1.56	−3.55	−1.56	−0.24	7.69	2.61	−1.70	10.18
Language (Semantics)	Cognition	0.34	2.26	9.32	−2.61	−3.10	−0.26	8.45	1.28	−7.80	16.77
Language (Speech)	Cognition	0.52	0.02	4.83	−2.79	−1.11	4.75	17.23	1.25	−6.12	10.24
Language (Syntax)	Cognition	−0.78	−1.28	−0.30	0.28	−1.66	−1.34	4.42	0.18	−1.48	5.54
Memory (Explicit)	Cognition	−1.62	−2.99	2.43	5.43	−7.48	−3.04	1.11	10.20	−2.50	12.24
Memory (Other)	Cognition	−0.20	−0.27	0.19	−1.28	−0.98	0.54	0.08	−0.74	−0.37	1.22
Memory (Working)	Cognition	−1.90	−2.21	9.02	−6.14	−3.77	6.58	−3.53	10.35	4.32	17.20
Music	Cognition	−3.04	−2.86	−1.75	−2.71	0.86	3.61	10.11	0.25	−3.01	0.72
Other	Cognition	−3.58	−3.09	−0.65	1.15	−7.32	−2.18	0.14	22.60	−1.30	6.01
Reasoning	Cognition	2.34	0.18	2.62	1.90	−4.43	−0.52	−2.90	4.85	2.18	8.40
Social	Cognition	−2.37	−3.72	−0.74	6.45	−4.41	−2.42	1.17	4.24	0.98	1.88
Soma	Cognition	0.48	0.17	2.39	0.35	−3.09	1.70	3.27	1.40	−1.42	1.14
Space	Cognition	3.26	0.44	13.82	−1.32	−1.58	4.58	−2.98	−1.18	−1.37	6.49
Time	Cognition	−0.60	0.52	0.66	−1.81	−0.65	1.07	0.98	1.13	0.80	2.29
Anger	Emotion	−0.45	−1.93	1.94	−2.53	−1.69	−1.70	3.85	0.98	−0.03	0.54
Anxiety	Emotion	0.29	−0.20	−0.66	1.22	−2.36	−0.55	1.69	5.29	−1.17	−0.57
Disgust	Emotion	0.22	0.94	2.77	0.37	−2.37	−2.24	4.64	3.02	−1.67	−0.30
Fear	Emotion	−1.35	0.35	4.28	−0.05	−1.86	−4.07	2.07	5.62	−3.31	−2.43
Happiness (Humor)	Emotion	−1.29	−1.48	3.26	0.28	0.02	−1.43	1.47	−0.20	−1.51	−1.38
Happiness (Other)	Emotion	−0.55	0.27	1.60	0.15	−2.86	−2.16	3.23	2.16	−2.26	−1.39
Other	Emotion	−4.70	−4.97	−2.35	3.47	−9.15	−4.27	2.74	25.52	−2.95	4.73
Sadness	Emotion	−1.08	−0.06	−0.48	−0.25	−3.40	−3.20	2.03	3.55	−2.12	−0.75
Air-hunger	Interoception	−1.31	−1.19	−1.22	−2.17	2.20	−1.55	0.36	0.61	−1.87	−1.51
Bladder	Interoception	−3.13	−1.45	−2.82	−2.37	1.07	2.32	1.53	2.77	−0.12	−1.85
Hunger	Interoception	1.05	0.51	1.83	−0.94	−0.05	−2.59	2.79	1.52	−2.28	−1.19
Other	Interoception	−2.43	−2.41	−1.75	−2.56	0.09	4.00	2.23	0.59	−0.74	−0.40
Sexuality	Interoception	−3.02	−0.09	4.73	−0.16	−4.20	−1.08	1.01	5.32	−4.06	0.67
Sleep	Interoception	2.69	−1.43	1.92	0.46	0.36	0.51	−0.82	1.08	−1.48	0.28
Thermoregulation	Interoception	1.21	0.75	−0.86	0.80	−1.33	−0.25	0.37	0.15	−1.02	−0.38
Thirst	Interoception	−0.53	−2.49	−3.18	0.81	−1.47	2.23	1.52	2.60	−1.07	−0.36
Audition	Perception	−4.34	−4.16	−4.30	−3.52	−4.80	2.31	21.30	0.44	−3.64	0.11
Gustation	Perception	−2.40	0.73	0.22	−2.83	−0.34	−1.37	3.00	5.79	−2.88	−0.72
Olfaction	Perception	−1.53	−1.52	−1.70	0.33	−0.99	−1.76	0.86	4.76	−2.00	−0.69
Somesthesis (Other)	Perception	−2.34	−2.46	−0.87	−4.51	−0.18	14.22	5.38	1.07	−0.63	1.03
Somesthesis (Pain)	Perception	−9.73	−4.34	−7.83	−5.32	0.02	8.45	10.00	11.53	1.19	−0.27
Vision (Color)	Perception	2.42	3.32	4.73	−2.58	−1.37	−1.05	−3.30	0.71	−0.23	1.90
Vision (Motion)	Perception	8.18	2.48	10.64	−2.91	−3.48	12.19	−5.75	1.58	−4.02	2.45
Vision (Other)	Perception	8.65	5.38	10.52	−0.93	−2.24	2.53	−1.10	−0.38	−1.98	2.81
Vision (Shape)	Perception	5.59	7.04	19.04	−4.16	−1.38	2.86	−3.33	−2.77	−2.94	4.29

Significant behaviors (z ≥ 3.0) highlighted.

ICA1-ICA3, visual areas with ICA1 medial, ICA2 OP and ICA3 lateral; ICA4, default mode network; ICA5, cerebellum; ICA6, sensory motor; ICA7, auditory; ICA8, executive control; ICA9 and 10, frontoparietal (ICA10 on the left).

Importantly, compared with the atlas study (Table 4), where significant “Perception:Vision” behaviors were reported in many anatomically defined regions, “Perception:Vision” behaviors were more restricted to classic visual areas in the resting state network study (ICA1-3; Table 5). This supports the notion of improved behavioral specificity for functionally derived vs. anatomically derived ROIs. For some networks, we observed a higher degree of significance across a wider range of behavioral domains than was expected. In particular, we observed several perceptual and motor networks that yielded significant cognitive domains, which informs as to the complexity of many-to-many mappings between brain regions and mental functions, as well as the potential risks associated with carrying out reverse inference (Poldrack, 2006; Chang et al., 2012; Yakoni et al., 2012) without a computational framework and underlying database structure. However, behavior analysis is intended to inform concerning a forward region-to-behavior relationship not the reverse.

## Discussion

The basic assumption of behavior analysis was that the spatial distribution of activation foci derived from the BrainMap database for each behavioral sub-domain represents the sub-domain's true probability distribution function. The large numbers of activation foci reported by some sub-domains indicate that this was a reasonable assumption (Table 1). We felt that large-N sub-domains (*N*_*b*_ > 1000 foci) would be reasonably represented but were concerned about lower-N sub-domains. However, integrated probabilities for the low-N sub-domains can be large if the ROI matches with the spatial distribution of activation foci. To test basic assumptions we formulated behavior-specific ROIs for each sub-domain and examined the behavior profile of each. To simulate a continuous PDF, desired for formulating ROIs, we smoothed each sub-domains PDF image using a 3-D Gaussian filter (FWHM = 10 mm). Behavior-specific ROIs were formed using a 25% threshold.

A highly significant *z*-score (*z* > 10) was seen in each behavior-specific profile for its paired sub-domain. In fact paired behavior sub-domains were ranked in the top four for all behavior-specific profiles (36 ranked 1st, 9 ranked 2nd, 5 ranked 3rd, and 1 ranked 4th). The “Action:Execution:Other” sub-domain had the highest *z*-scores for behavior-specific ROIs of several other Action sub-domains (Action:Imagination, Action:Motor Learning, and Action:Preparation). This was not unexpected since Action:Execution is a behavior that occurs in conjunction with these behaviors and would be designated as such in BrainMap. Interestingly, “Emotion:Other” had the highest *z*-score for the behavior-specific ROIs from “Interoception:Sexuality” and “Perception:Olfaction” behavior sub-domains. This suggests that a strong emotional response in experiments with these sensory driven behaviors. Importantly, all other behavior-specific profiles from the low-N Interoception domain, paired best with their sub-domain. This evaluation showed that a behavioral sub-domain can be significant when an ROI that matches the spatial distribution of activation foci for the sub-domain, even for low-N sub-domains.

The regional benefit of behavioral analysis obviously diminishes for larger ROIs, so we recommend using ROIs smaller than cerebral hemispheres (Table 2). Effect size decreases as the ROI size increases, approaching zero for a whole brain ROI, as both *p*_*o*_ and *p*_*e*_ approach unity (Equation 1). We expect that likely use scenarios will be with ROIs formulated from statistical or anatomical maps, which are much smaller than cerebral hemispheres. There are several factors that relate to significant behaviors for such ROIs. The variance in effect size (*p*_*o*_−*p*_*e*_) is inversely related to *N*_*b*_, such that larger-N sub-domains (Table 1) can have higher *z*-scores for the same effect size. Therefore behavior analysis for low-N sub-domains, such as Interoception, may require a larger effect size to reach the same significance level. Adding more studies to the BrainMap database for low-N sub-domains will help balance this effect and is a recommendation of this project. An effect size approaching +1 (*p*_*o*_→1 and *p*_*e*_→0) indicates a good spatial match between the distribution of activation foci for a behavioral sub-domain and the ROI, and also leads to a reduction in variance, since variance of both *p*_*o*_ and *p*_*e*_ approaches to zero at these extremes (see Equation 1). This non-N related variance property indicates that the *z*-score for a low-N sub-domain can be significant for larger effect sizes. These factors, increasing the numerator of Equation 1 and the decreasing the denominator, can jointly lead to high *z*-scores for sub-domains with “highly localized” activation foci when probed using an ROI that fits the location and extent of the sub-domain.

While the regional specificity increases with smaller ROIs, the utility of automated behavior analysis also diminishes with diminishing ROI size. Unlike the Talairach Daemon (Lancaster et al., 2000) it is not reasonable to use behavior analysis for single coordinates. However, it is possible to estimate behaviors in the neighborhood of a single location. This can be achieved by varying the search range about a point ROI, at an *x*-*y*-*z* coordinate, until significant sub-domains are reported or until the range reaches some user defined limit. If no significant sub-domains were found within the range limit the reported outcome would be “no significant behaviors within this neighborhood.” This approach has not yet been tested, but a future release of the behavior analysis software will support using a table of coordinates to append a list of significant behaviors within a neighborhood, similar to what is done for the Talairach Daemon for anatomical labeling of locations.

The BrainMap database changes almost daily as new papers are entered. Periodic updates to the 4-D behavior image will be provided to keep pace with changes. Database growth was experienced during the development of the behavior analysis application where the 4-D behavioral image was updated several times to improve on low-N sub-domains.

**Figure 4:**
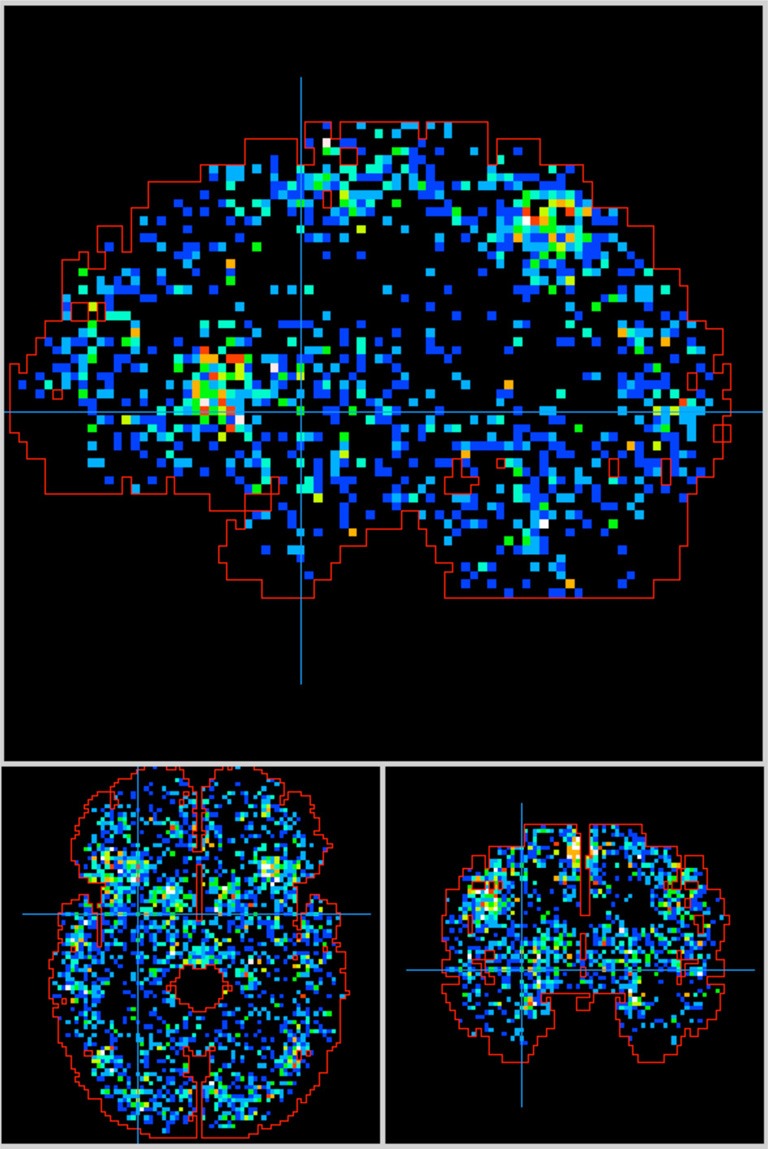
**Activation foci from all 51 behavior sub-domains are distributed throughout the Talairach brain.** Outline from the Talairach Daemon. Crosshair at (−28, 0, 0).

The null hypothesis was that the observed probability *p*_*o*_ was not different from the probability expected *p*_*e*_ if activation foci were randomly distributed throughout the brain, i.e., not localized. There is a potential problem with the way *p*_*e*_ was calculated, based on whole brain volume, since activation foci should not fall within all spaces within the brain, i.e., white matter or ventricles. This suggests that we should use a volume less than whole brain when calculating *p*_*e*_. However, as illustrated in Figure 4 the distribution foci throughout the brain includes these regions, albeit with fewer foci/volume. This is due to many effects, including differences in brain normalization methods, differences in resolution of functional images, and differences in methods to determine locations of activations. To avoid making assumptions that might vary over time we felt that the best alternative was to assume that for the null case any location within the brain would be equally likely for activation. This method worked well to delineate significant behaviors in this project. Also, this approach worked well for ROIs placed in WM, where most *z*-scores were negative, and no significant behaviors were indicated. Finally, this approach has worked well for rejection of ICA components associated with motion artifacts (mostly at brain boundaries), where most *z*-scores are negative, and no significant behaviors are indicated.

The automated regional behavioral analysis application is a software tool that provides real-time access to the comprehensive set of behavioral data in the BrainMap database. The only user action is to define the ROI in a spatially normalized brain image. To simplify interpretation, results are presented as a behavior profile chart with a table of behaviors ranked by *z*-scores. Benefits of automated regional behavior analysis were demonstrated in brain atlases, in individual and group fMRI studies, as well as for resting state networks. The behavioral analysis software provides a novel approach to organize, share, and interpret database information and, as such, should provide a unique resource for the neuroimaging community.

### Conflict of interest statement

The authors declare that the research was conducted in the absence of any commercial or financial relationships that could be construed as a potential conflict of interest.
